# Contraceptive behaviors and media influence among women in Bangladesh: exploring the effects of age and education

**DOI:** 10.3389/fgwh.2025.1492105

**Published:** 2025-05-16

**Authors:** Monira Parvin Moon

**Affiliations:** Department of Rural Development, Gazipur Agricultural University (GAU), Gazipur, Bangladesh

**Keywords:** contraceptive behavioral practices, media exposure, age effects, education effects, Bangladeshi women

## Abstract

**Background:**

Married Bangladeshi women of reproductive age have improved their contraceptive use in recent decades. This review study searched PubMed, Scopus, and Google Scholar to examine how age and education affect Bangladeshi women's contraceptive behaviors.

**Results:**

Modern approaches were most used by 15–19-year-olds. Between 2011 and 2017–2018, fewer educated women read a newspaper or magazine at least once a week, suggesting older women watch more TV. Over time, elderly adults utilize none of the three media at least once a week. According to the findings, elderly women are the least likely to receive weekly media coverage of their contraceptive treatments. In short, the study found that younger women utilize current contraceptive techniques, while older women may employ them less frequently, and better education significantly enhances contraceptive use, as there is a strong correlation between educational attainment and the utilization of contraception. Moreover, the results indicated that women who were not exposed to the media were less likely than those who were to use contemporary techniques.

**Conclusion:**

The report strongly recommends improving the education of disadvantaged people, particularly Bangladeshi women. To boost the nation's usage of contraceptives, women need effective health behavior education, family planning, and counseling programs.

## Highlights

• There are age-related influences on contraceptive use among women.
• Contraceptive methods among women are influenced by education.
• Media influences women's use of contraception in Bangladesh.

## Introduction

Contraceptive behavior is a significant public health concern globally, especially for women (1–3). The term “contraception” originally referred to a method of birth control (4). Rahman et al. (5) and Haq et al. (6) found contraception effective for family planning and fertility control, but approximately 200 million women worldwide struggle with safe and effective methods. Bangladesh's contraception prevalence rate (CPR) has plateaued, as, having increased to 61.0% in 2011, it was 62.4% in 2014 and 62.0% in 2017 (7). Subsequently, increasing the prevalence of contraception in Bangladesh from 62% to 75% by 2020 was one of the primary goals (8). Reproductive health outcomes in underdeveloped nations are also influenced by a woman's capacity for decision-making (9–11). Bangladesh has made progress in health and family welfare, but reproductive and sexual healthcare access remains challenging, with increased contraceptive use being a goal of various organizations and sectors (5, 6). To achieve this, 69 of the world's poorest countries established the Family Planning agenda in the wake of the 2012 London Summit on Family Planning. The agenda aims to provide 120 million more women and girls in 69 of the world's poorest countries with access to family planning information, services, and supplies by 2020 (8, 9). Contraception is now used by 64% of married or cohabiting women worldwide between the ages of 15 and 49, but it is only used by 40% in the least developed countries (12, 13).

Bangladesh has made notable progress in terms of the percentage of married women of reproductive age who take contraceptives, increasing from 8% in 1975 to 62% in 2014 (14). Further benefits include reducing health risks related to pregnancy and maternal death, improving perinatal outcomes for newborns and infant mortality, empowering individuals, and enhancing education when population growth and contraceptive use are reduced (15, 16). Contemporary forms of contraception encompass oral contraceptive pills, implants, injectable contraceptives, contraceptive patches, rings, intrauterine devices (IUDs), and condoms. The conventional contraceptive methods encompass the rhythm technique and coitus interruptus (15). These persistent public health issues in low- and middle-income countries (LMICs) significantly contribute to the increased prevalence of unplanned pregnancies, shorter intervals between pregnancies, and a greater rate of pregnancy terminations (17). The availability of contemporary contraceptive methods, as opposed to the absence or use of traditional contraceptive methods, can effectively tackle these concerns by empowering women to exert autonomy over their reproductive decisions, allowing them to space pregnancies and strategically schedule childbirth (18). Bangladesh's prevalence of contraceptive use (62%) is higher than that of other South Asian countries, such as India (58%), Nepal (50%), Pakistan (35%), and Afghanistan (23%), but it is still very close to the global prevalence of contraception (64%). The usage of contraceptives by Bangladeshi women was likewise linked to a preference for boys (19).

Previous studies conducted in Bangladesh have found that several sociodemographic considerations, such as age, education, and the wealth of the household, are strongly linked to contraceptive use (6, 20). Although previous research has examined the determinants of modern contraceptive use, only a small number of studies have considered both traditional and modern approaches (6, 21, 22). Nevertheless, contraceptive use, as a binary outcome variable, was analyzed using binary logistic regression in these investigations. Understanding the factors associated with the use and selection of contraceptive techniques is crucial, as the current use of these methods is suboptimal (20). Bangladeshi women use both modern and traditional contraceptive methods, highlighting the need for a deeper understanding of the factors influencing their use (22, 23). Furthermore, because a high contraceptive prevalence rate is always expected for birth control in densely populated areas like Bangladesh, it is vital to periodically assess prevalence and risk factors to monitor the current situation. Consequently, this study will help to fill the gaps in the literature. Therefore, this study investigates the prevalence of contraceptive behaviors and media influence among women in Bangladesh, with the effects of age and education from the Bangladesh Demographic and Health Survey (BDHS) (24–26).

## Methodology

### Search strategy

Following PRISMA criteria, the study used data from the 1993 to 2024 Bangladesh Demographic and Health Surveys and searched PubMed, Scopus, Google Scholar, and peer-reviewed publications for related studies (27). The study searched for articles on Bangladeshi women's contraceptive habits and media influence based on age and education.

The key search terms for this study were as follows: “Reproductive Health,” “Contraceptive use,” “developing countries,” “contraception,” “family planning,” “Women Autonomy and fertility,” “Decision-making Autonomy,” “Fertility Preferences,” “Media exposure to contraception,” “systematic reviews,” and “Bangladesh” (Table 1).

**Table 1:** Relevant studies on contraceptive behaviors among women in Bangladesh.

Study	Study aim	Age (years)	Sample size	Study design	Key result	Exclusion	Study quality
Rana et al. (28)	Examine the trends and determinants of modern contraceptive uptake among later reproductive-aged women in Bangladesh	≥35	BDHS (25, 29) 17,736	Multilevel logistic regression model	Modern contraceptive method uptake was higher among women who reported exposure to mass media compared to women who reported no exposure to mass media	Women who are currently pregnant	High
Rana et al. (30)	Examine the trends and determinants of modern contraceptive practices among late reproductive-aged women in Bangladesh	35–49	17,736 women	Multilevel logistic regression model	The probability of using modern contraceptive methods exhibited a notable decline in relation to increasing age, the educational level of women's partners, and their categorization within the richer or richest wealth quintile	Women who were pregnant	High
Alam et al. (31)	Prevalence and determinants of adolescent childbearing	15–19	2,023 (29) 1,951 (24)	Univariate and multivariate logistic regression	Adolescent childbearing prevalence rate was 30.8% in 2014 BDHS and 27.6% in 2017–2018 BDHS	Women who mentioned that they had never had sex.	High
Chowdhury et al. (32)	Fertility and reproductive health have expanded	15–49	212,271 (from several research participants)	Systematically screened	Negative relationship between women's empowerment and the control of fertility and reproductive health	Studies not reporting women empowerment factors of fertility	Average
Sharif et al. (33)	Factors associated with the permanent and long-acting reversible contraceptive (LARC) method use compared with short-acting reversible contraceptive (SARC) methods	15–49	9,669 (24)	A multilevel multinomial logistic regression model	Women with no or less education, non-Muslims, and having parity of ≥3 were more likely to use both permanent and LARC methods than SARC methods	Those who were not using any contraception method and those who were using a traditional contraception method	High
Kundu et al. (34)	Determine the prevalence of using modern and traditional contraceptive methods and the factors that explain the use of contraceptive methods	15–49	11,452 (weighted) (24)	Multilevel multinomial logistic regression	Highlights the importance of male partners’ decision-making regarding women's contraceptive use	Those who had never had sex or sexually inactive in the last 4 weeks	high
Roy et al. (35)	Examine the socioeconomic, demographic, and other critical factors linked to the use of family planning (FP) in the studied areas during the COVID-19 pandemic	15–49	423	A multivariate logistic regression model	Higher rate of contraceptive use	Those with mental and severe health problems, those who were pregnant, widowed, or divorced	High
Majumder and Khan (36)	To determine the factors that connect contraceptive use as well as gathering knowledge of family planning (KFP) of indigenous women	15–49	223	Binary logistic regression analysis	Current contraceptive use among indigenous women was significantly influenced by the location of residence, the education of their spouse, maternal healthcare service in the local area, and the distance of the health service center from home	Those who were not using any contraception method	High
Islam et al. (37)	Variations in contraceptive use	15–49	15,699 (29)	Multivariate logistic regression models	There were significant differences in divisional variations in contraceptive use in rural areas	Women who mentioned that they had never had sex.	High
Hossain et al. (21)	Socioeconomic, demographic, and others key factors that influence the use of contraception in Bangladesh	15–49	16,858 (29)	A mixed effect logistic regression model	Selected independent variables were significantly associated with contraception use in Bangladesh	Ever married (included currently married)	High
Islam (38)	Couples’ joint participation in household decision-making and modern contraceptive use (MCU)	<25	3,507 (25)	Binary logistic regression	Couples’ joint decision-making power on women's healthcare and children’s healthcare, and visiting family members or relatives emerged as the third most influential factor that might be associated with MCU	Pregnant fecund women	High
Islam et al. (39)	Association between sociodemographic factors and contraceptive use among fecund women	<25	3,744 (24)	Binary logistic regression	Husband-wife joint participation in decision-making on healthcare increases the likelihood of using contraceptives	All married women	Average
Biswas et al. (40)	Whether a woman's autonomy matters or not in determining her ability to exercise reproductive rights in rural Bangladesh	15–49	200	Bivariate linear regression technique	Women's autonomy status is strongly associated with their ability to exercise reproductive rights status	Women who do not have a child	High
Islam and Hassan (22)	Effects of women's knowledge, attitude, and family planning approval on contraceptive use of married women	15–49	430	Path analysis	Media exposure significantly affects family planning approval, increases the positive attitude toward contraceptives, and significantly increases the knowledge of contraception	Not mentioned	Average
Rahman et al. (41)	Women's decision-making autonomy and contraceptive behavior	15–40	8,456 (42)	Multiple logistic regression	Women's autonomy to increase contraception	Women who are currently pregnant	High
Gazi et al. (43)	Assess changes in knowledge among married women of reproductive age on selected reproductive health issues	13–49	54,116	Simple random sampling method	Investment in the reproductive health sector, particularly in existing government programs, improves rural health (RH) outcomes	Not mentioned	Average
Goni and Rahman (44)	Assess the impact of education and media on contraceptive use, and also identify the factors that are associated with the current use of contraception and continuing use of contraception	15–49	10,146 (42)	Univariate and multivariate techniques	Contraceptive use was higher among educated women and those women who watched TV at least once a week as compared with their respective counterparts	Those who were divorced, widowed, or not living together	High

### Research questions identification

**Figure 1:**
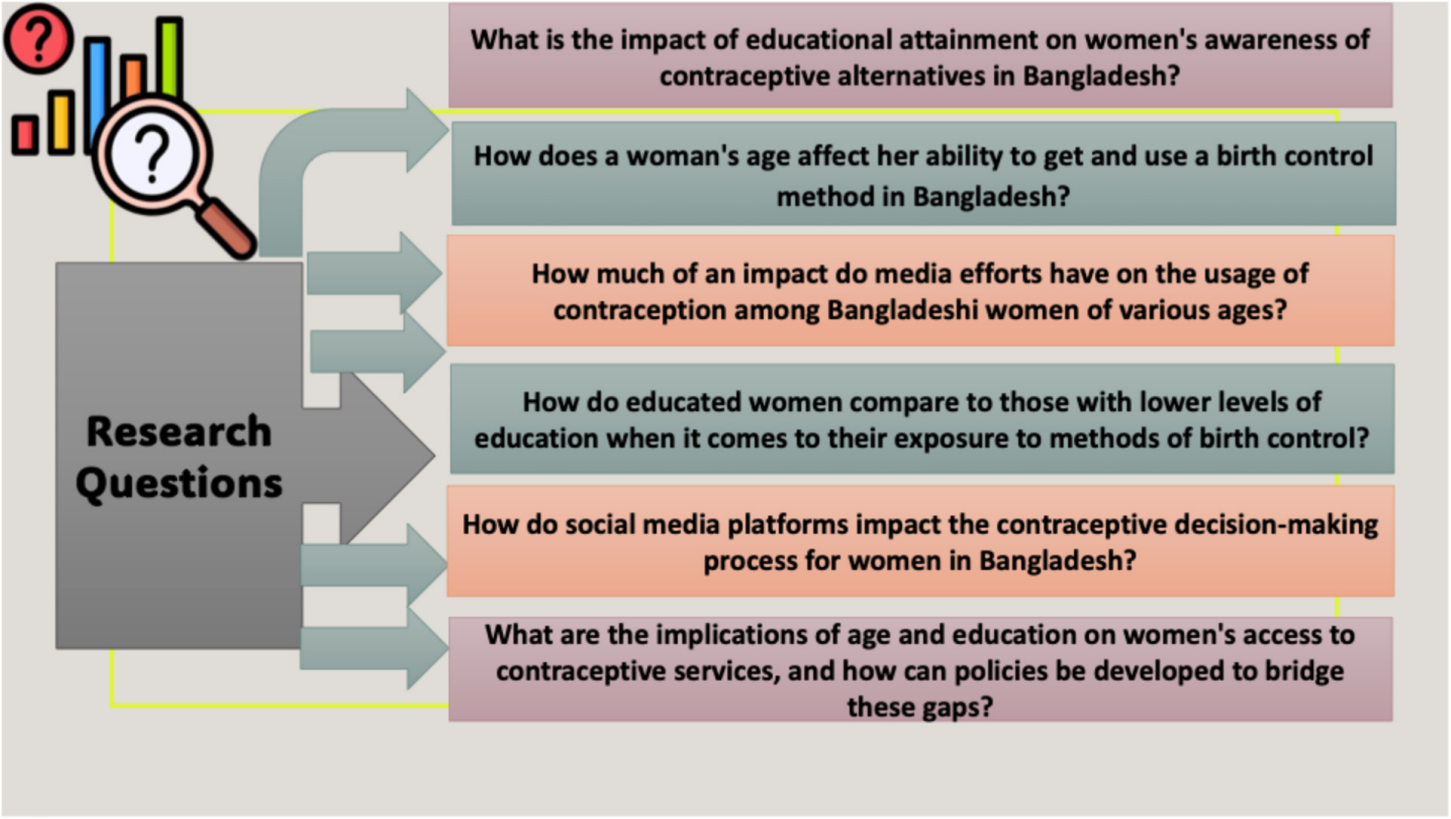
Research questions for the study.

Iterative research idea generation was described by Arksey and O'Malley (45). The research questions have been presented in Figure 1, which will lead this study.

### Identification and screening

**Figure 2:**
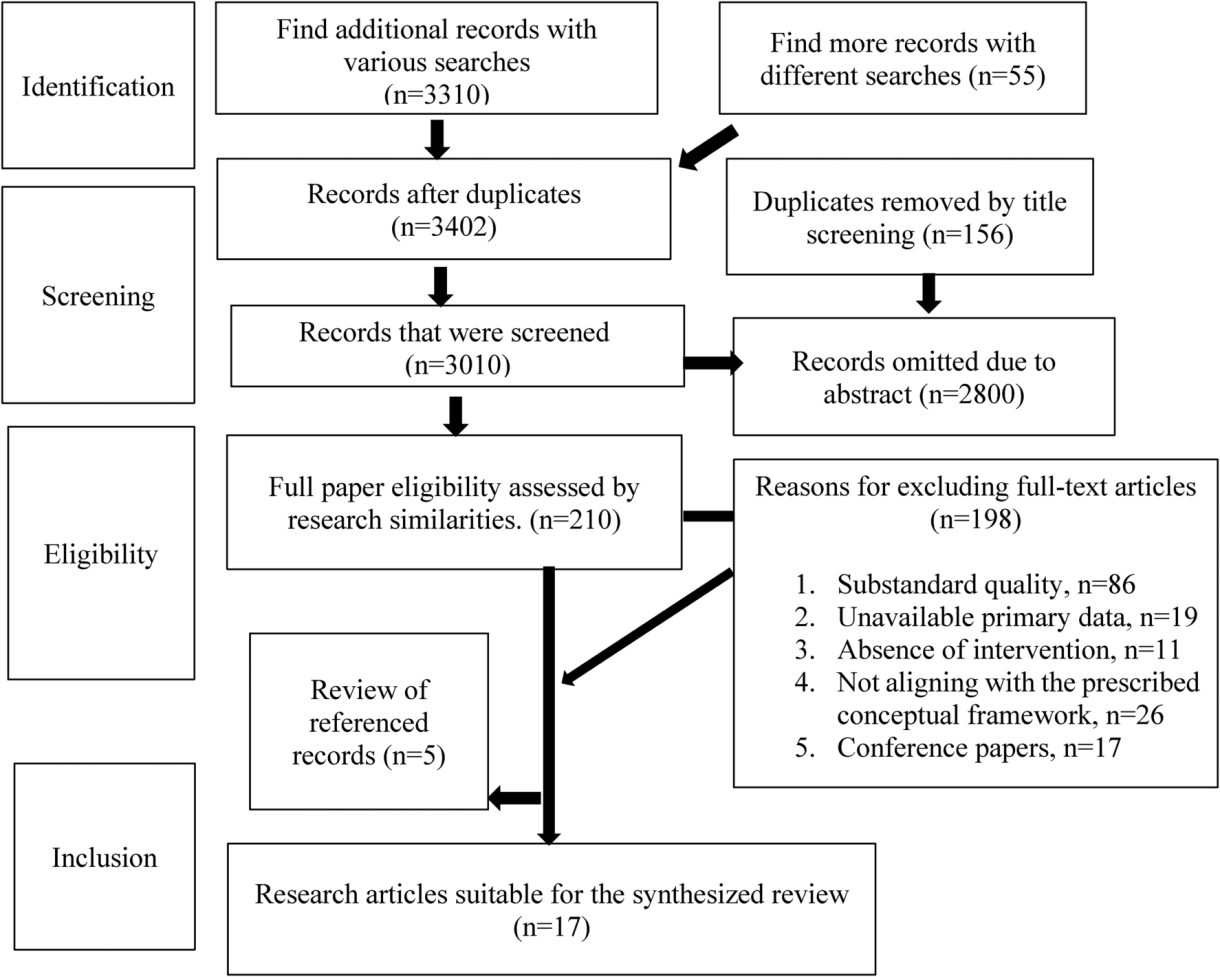
PRISMA flowchart for the systematic review.

The reference lists of every article that was chosen were also examined. A total of 3,310 papers were initially identified through the systematic search and an additional 55 articles were discovered by searching different databases. Among the 3,010 publications that were assessed, 2,900 were excluded after abstract screening. A total of 210 full-text articles were evaluated to determine whether or not they were eligible for inclusion in this study. Five such articles were chosen to be included in the review of references. Following careful consideration of a number of aspects, a final selection of 17 articles was made for additional research. In total, 198 full-text articles were disregarded for a variety of reasons, including but not limited to: substandard quality (86), unavailable primary data (19), absence of intervention (11), not aligning with the prescribed conceptual framework (26), and being conference papers (17). After removing 198 full-text articles, the study identified 12 articles that were incorporated into the review of cited records (5). Ultimately, 17 pertinent publications were selected for the synthesized review (Figure 2). Table 1 describes the 17 studies on contraceptive behaviors among women in Bangladesh, along with the aim, age group, sample size, study design, key results, exclusions, and study quality. The majority of the noteworthy results were linked to the following: women's autonomy status is strongly associated with their ability to exercise their reproductive rights status; contraceptive use was higher among educated women; and media exposure significantly affects family planning approval, increases positive attitudes toward contraception, and significantly increases knowledge about contraception.

### Study selection criteria

For current studies of contraceptive behaviors and media influence among women in Bangladesh, we used extensive inclusion and exclusion criteria. The review included studies on women and excluded studies that included men. In addition, reports from other developing and developed nations are included to allow for comparing the actual pictures of the contraceptive behaviors and media influence among women. In addition, only articles written in English have been included.

### Extracting data

The extraction process was carried out using data from the 1993 to 2024 Bangladesh Demographic and Health Surveys and from searches of PubMed, Scopus, Google Scholar, and peer-reviewed publications for related studies. The PRISMA method has been used to explore the contraceptive behaviors and media influence among women in Bangladesh.

## Results and discussion

**Figure 3:**
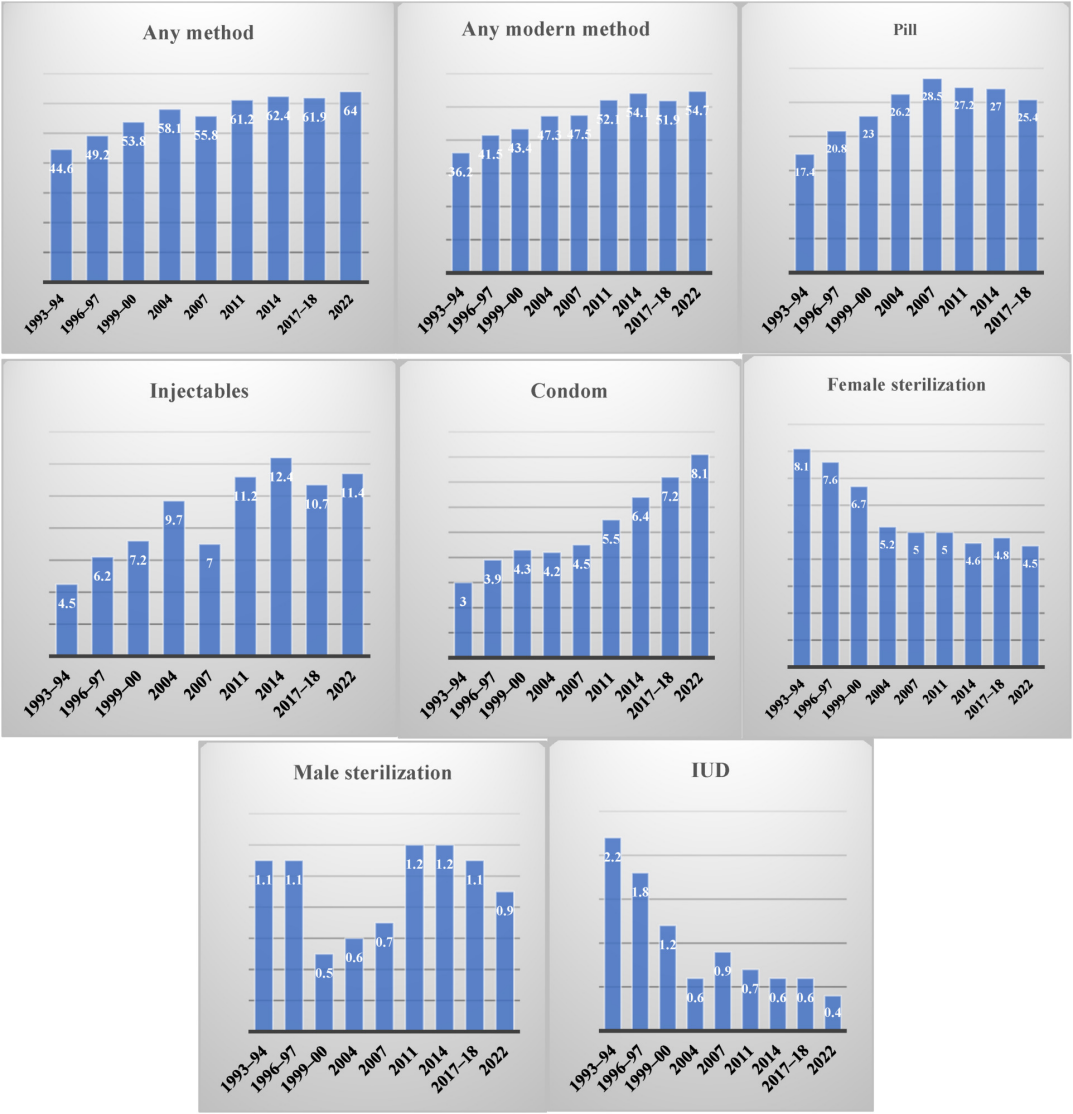
CPR from 1993 to 2022 in Bangladesh.

The CPR increased slightly from 61.2% in the 2011 BDHS to 62.4% in the 2014 BDHS, according to the BDHS data (14). After controlling for other variables, Kibria et al. (46) found no link between the use of contraceptives and a variety of variables, including the working status of women and spouses’ education level. This finding is significant because earlier studies (47, 48) have found a relationship between these characteristics and the use of contraceptives. Figure 3 represents the contraception prevalence rate from 1993 to 2022, showing eight types of practices, including any method, any modern method, pill, injectables, condom, female sterilization, male sterilization, and condom. The study showed that the prevalence of any method (64%), any modern method (54.7%), and use of condom practices (8.1%) significantly increased from 1993 to 2022 in Bangladesh. Using the pill (highest 28.5%), injectables (highest 12.4%), and male sterilization had fluctuations. In contrast, using female sterilization (highest 8.1%) and IUDs (highest 2.2%) had a significant decrease over the years.

### Age-specific CPR currently in use

**Figure 4:**
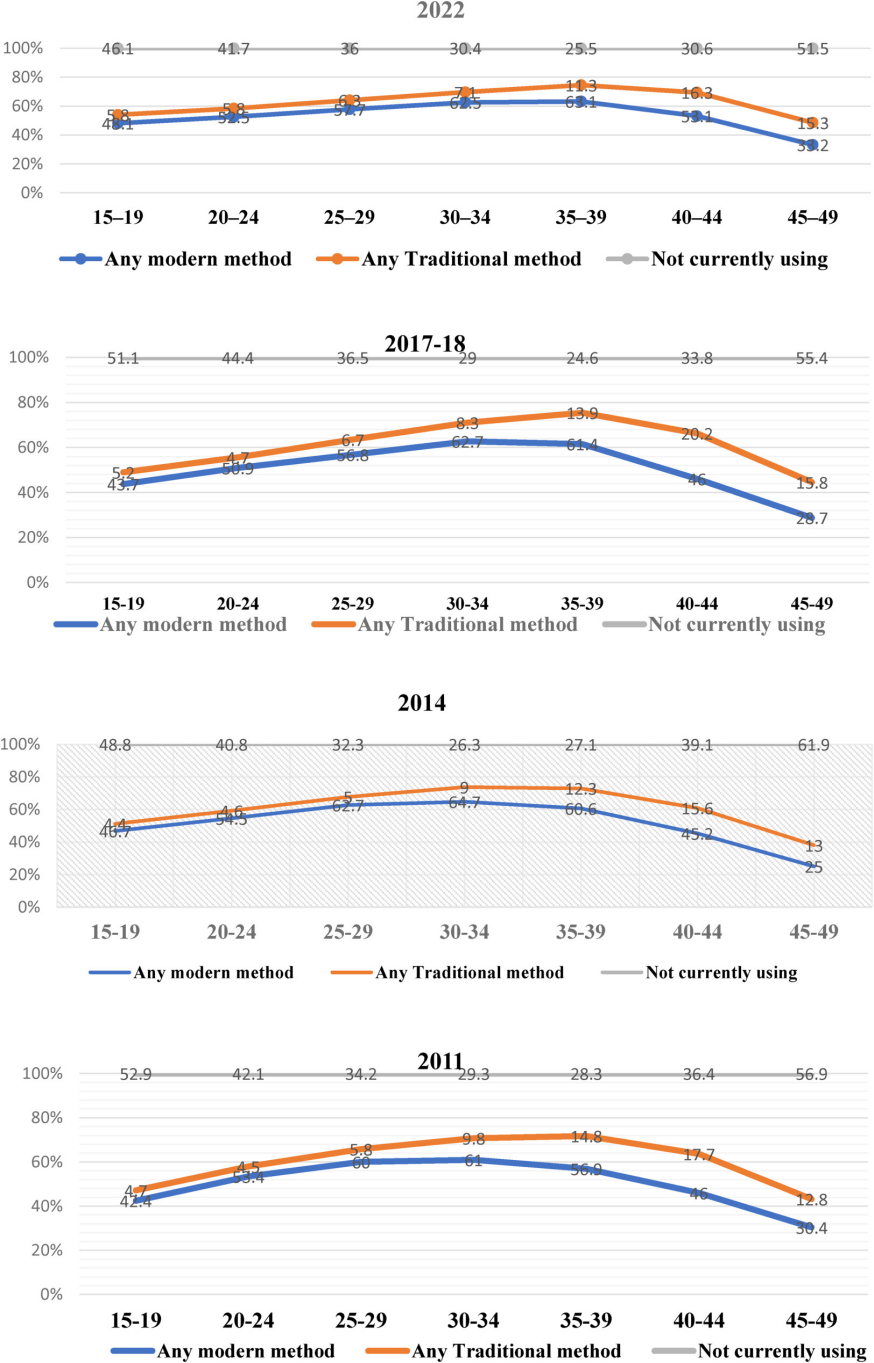
Age-specific CPR currently in use from the BDHS (24–26, 50). The proportion of married women aged 15–49 using contraceptives by age.

The probability of using CPR decreased as age increased and rose as age at marriage increased among fertile women under 25 years old. Hence, the creation of service centers accessible to young individuals in suitable locations and the provision of necessary resources would effectively promote the utilization of reproductive health services among young people (49). Figure 4 shows the data for current use of contraception by age using the data from BDHS (24, 25, 29, 50), showing three categories including any modern method, any traditional method, and not currently using. The CPR was 64% among married women aged 15–49, with 55% using modern techniques and 9% using traditional methods. In 2022, any modern method and any traditional method had a fluctuation. The prevalence of any modern method was highest in the age group 15–19 in 2022 (48.1%), and it was lowest for the age group of 35–39, while it had a significant decrease in the 45–49 age group. However, the prevalence of any traditional methods was highest for the 45–49 age group (15.3%) and lowest for the 15–19 age group (5.8%). Similarly, for 2017–2018, 2014, and 2011, this study found that the prevalence of the use of any modern methods was higher among the 15–19 age group, while the use of any traditional method was the lowest in this age group. A common explanation for why fewer older women were using birth control is their declining fertility, whereas fewer younger women using it is often associated with their desire to have more children (44). Among women aged 25–34, the use of modern methods of contraception was most prevalent, with almost two-thirds of women reporting doing so (34). This study's results are consistent with those of a previous study by Forty et al. (51) that found that adolescent girls and women are more likely to plan to use contraception. Haq et al. (6) also found that the usage of contraceptives decreases with age, which suggests that older women are less likely to utilize contraceptives than their younger counterparts. Similar to the findings of another study that has already been conducted (52), this kind of association between age and the use of contraceptives has been identified. Among women of all age categories, the pill was the most commonly utilized contraceptive technique, with the exception among those aged 45–49, who were more inclined to practice intermittent abstention. Research by Hossain et al. (21) revealed a comparable trend among women aged 30–49 who had a higher likelihood of undergoing sterilization compared to younger women. In a cross-sectional study conducted in the Narsingdi District in the Dhaka division, Islam (53) similarly documented comparable findings, indicating that the prevalence rate of contraception was lower among younger women as compared to older women. Contraception may be used less frequently by older women because they are less likely to engage in sexual activity or have decreased the frequency of their sexual encounters. In contrast, younger women may not desire to have children at such a young age (51).

Numerous studies have shown that the use of contraception can significantly decrease fertility rates and eventually enhance the health of both mothers and children. However, the rate of not using any contraception was constant over the periods of 2022, 2017–2018, 2014, and 2011. The study conducted by Rana et al. (28) revealed that over 54% of women aged 35 and above do not utilize contemporary contraceptive techniques. Furthermore, there have been no notable changes in their utilization during the years investigated. The likelihood of modern contraceptive method adoption was shown to be lower among women aged 40–45 and 45–49 compared to those aged 35–39. According to Kundu et al. (34), the prevalence of contemporary contraceptive techniques among women in Bangladesh was 72.16%, while traditional methods were utilized by 14.58% of women. The reluctance to utilize modern contraceptive techniques was higher among older women (35–49 years) compared to women in the 15–24 years age group.

### Present status of CPR in the field of education level

It has been found that when women have access to quality education, they are more likely to be receptive to novel family planning methods (54). Furthermore, women who have completed a greater number of years of schooling are more likely to have professional or other forms of work, which may cause them to be less inclined to have a large family. Among Bangladeshi women, those with higher levels of education were more likely to use birth control than those with lower levels of education (49). Furthermore, the results indicate that women who had completed elementary school education had a greater likelihood of utilizing contraceptives compared to their uneducated counterparts. These findings align with prior research that has demonstrated a comparable correlation between educational level and contraceptive usage (55–58). Education empowers women to make independent decisions regarding fertility-related issues and also enhances their ability to exercise their reproductive health rights, in contrast to women who have received limited education. Furthermore, women who have acquired an education are likely to have a more thorough understanding of the benefits of using contraception to reduce unintended pregnancies compared to women who lack education. Thus, it is crucial for family planning service providers to give priority and address the needs of women who have little or no education during family planning interventions. Such would empower individuals to obtain essential knowledge on reproductive health, hence improving their acceptance of contraceptive methods.

**Figure 5:**
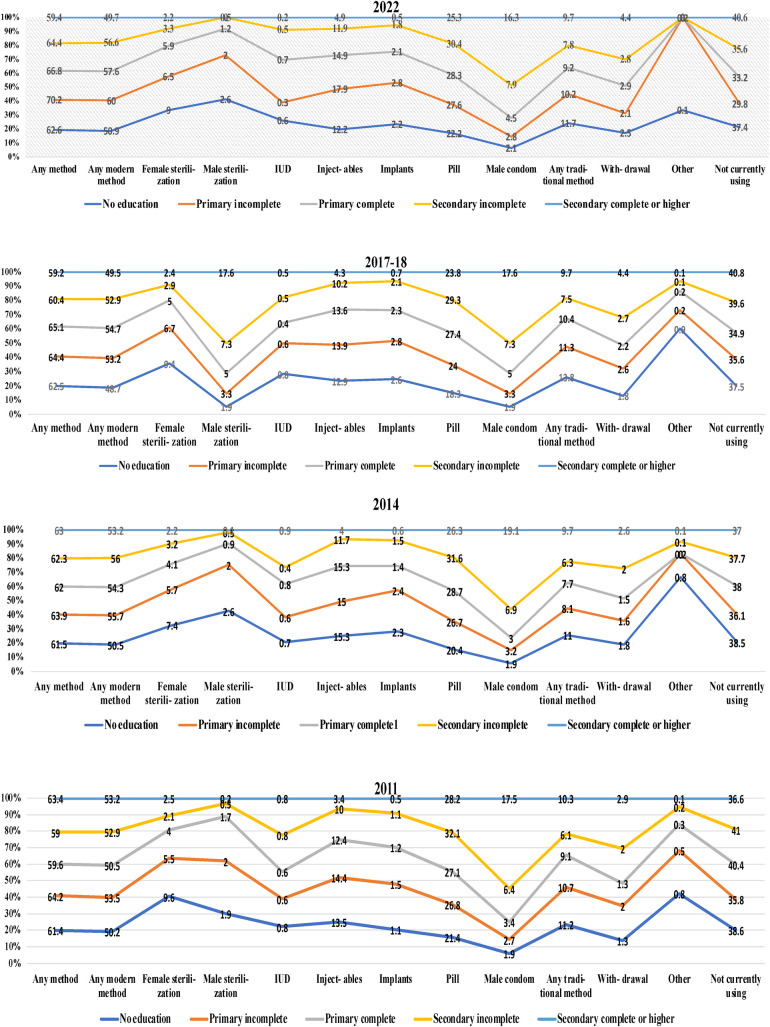
Present status of the CPR by education. Distribution of current contraceptive methods among married women in Bangladesh in 2022, 2017–2018, 2014, and 2011 according to education data from the BDHS.

Figure 5 shows the current use of CPR by education over the years from the BDHS (24, 25, 29, 50). The figure indicates that the use of any contraceptive method among women with no educational background in the years of 2011, 2014, 2017–2018, and 2022 was 61.4%, 61.5%, 62.5%, and 62.6%, respectively. Among the respondents with a primary education, the prevalence of any modern method, male sterilization, implants, and withdrawal increased. A prior study (20) provided evidence that young girls and women were more inclined to intend to use contraception, which supports the results of our study. Furthermore, Haq et al. (6) examined the correlation between women who had received an elementary education and those with secondary and higher levels of education, and their propensity to take contraceptives, in comparison with women with no education. A reason for this is that an increased level of education can offer the chance to acquire more knowledge about contraceptive methods and ensure improved availability of services. However, the prevalence of using an IUD, injectables, male condoms, any traditional method, other methods, and not currently using contraception among the respondents with incomplete primary education decreased.

The percentage of individuals in the complete primary education group who used any technique, any modern method, female sterilization, injectables, pills, male condoms, and withdrawal while they were using an IUD increased, whereas the percentage of individuals who used any method decreased. However, the use of any traditional method and other methods did not change. Among those with a secondary education, the use of any method, any modern method, female sterilization, male condom, any traditional method, and withdrawal increased from 2011 to 2022, while the use of male sterilization, IUD, injectables, and pills, and not currently using a contraception method decreased, and others were constant from 2011 to 2022. Ultimately, data revealed that possessing a secondary education or higher maintained a consistent perspective on activities across the years. Thus, from this figure, we can depict that the current use of contraception has increased over the years due to education. In the research carried out by Hossain et al. (21), it was discovered that 72% of the individuals who took part in the study were employing modern methods of birth control. This is an increase from the 62% of women in Bangladesh who were using these methods in 2014. The utilization of conventional methods of birth control has also reached 14.6% in this survey, which is in accordance with the present study. In 2014, this percentage was just 8.1%. According to Hossain et al. (21), in 2014, over 37% of Bangladeshi women did not make use of any method of birth control. However, according to recently conducted research, this figure has dropped to 13%. To achieve a contraceptive prevalence of 75% by the year 2020, the Health, Population and Nutrition Sector Development Programme (HPNSDP) of Bangladesh established strategic objectives to improve the overall utilization of family planning. These objectives were designed to ensure that family planning services are accessible, affordable, and acceptable to all men and women who are in their reproductive years (8, 59).

Education, contraception use, and reproductive practices are linked, and highly educated women are more active in childbearing decisions (60). Kabir et al. (61) found that women with higher education used contemporary contraceptives more, rising by 3% over time. Over time, women's employment status, husband's education, number of live children, and fertility desires decreased modern contraceptive use. Illiterate women used contraception less than educated women. Higher education greatly boosts contraception use (62, 70). Educational achievement is strongly connected with contraceptive use. Fleischer (63) states that a woman's education affects fertility management and contraceptive use. Women with higher levels of education are more likely to use contemporary contraception, according to research by Hossain et al. (64) and Pazol et al. (65). They concluded that women have more control over their reproductive health, family planning decisions, and access to contraception when they receive more education. More access to education, particularly in rural areas, could help reduce the population of Bangladesh by increasing the number of women who utilize contraception. There is evidence from research conducted by Gurmu and Tariku (66), Howlader et al. (67), and Tamirat et al. (68) that suggests that women who have a higher level of education are less likely to engage in risky reproductive behaviors in comparison to women who have a lower level of education. Educated women utilize modern birth control more than uneducated women because more educated women know about and can use modern contraception (69).

### Exposure to various forms of mass media with CPR

**Figure 6:**
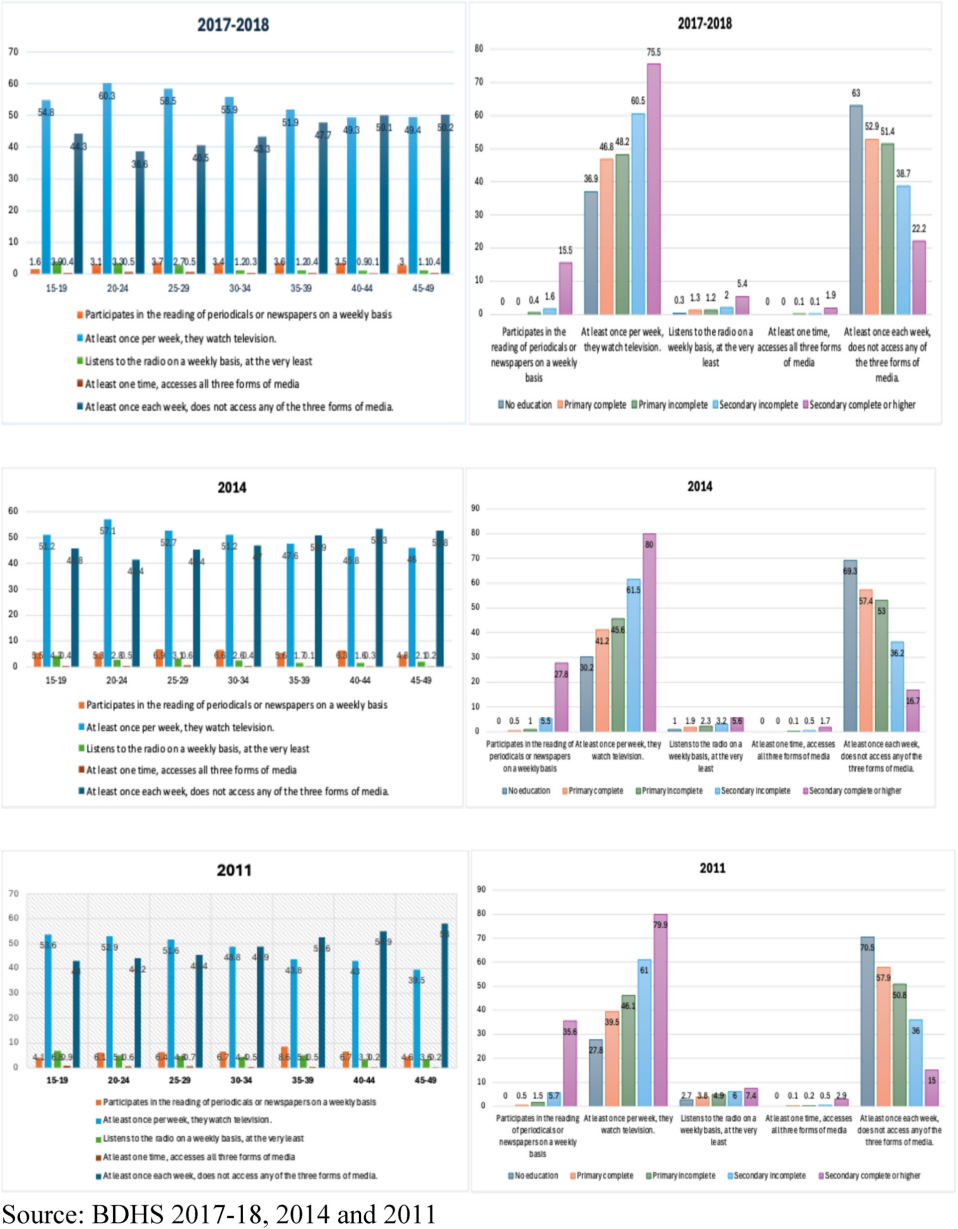
Exposure to mass media. Proportion of never-married women (15–49 years old) who, based on their age and level of education, were exposed to particular media on a weekly basis.

Exposure to mass media, such as radio and television, has an important influence on reproductive behavior. Figure 6 illustrates the percentage of ever-married women aged 15–49 who were exposed to specific media on a weekly basis according to age and education from BDHS (24–26). The figure shows that women aged between 25 and 29 read a newspaper or magazine at least once a week, which is the highest, while women aged between 15 and 19 had the lowest percentage at 3.7% and 1.6% for 2017–2018, respectively. The findings of Haq et al. (6) showed a noteworthy correlation between the utilization of contraceptive methods and being exposed to family planning information in the media. In each of the three studies, the findings indicated that there was a large amount of exposure to messages about family planning through the various kinds of mass media. This finding is consistent with the findings of previous research carried out in Bangladesh (85). Women who had no exposure to mass media were less likely to employ modern methods than those who had exposure to media (49). This was the case among the respondents who were asked about their usage of modern methods.

This study found that women aged 25–29 and 35–39 read a newspaper or magazine at least once a week, which is the highest, while women aged 45–49 and 15–19 had the lowest percentage at 6.9% and 4.8%, and 8.6% and 4.1% for 2014 and 2011, respectively. Women with a complete secondary or higher education had the highest prevalence of reading a newspaper or magazine at least once a week at 15.5%, 27.8%, and 35.6%, respectively. Thus, the prevalence of women with higher education reading a newspaper or magazine at least once a week decreased from 2011 to 2018. Moreover, the prevalence of watching television at least once a week was higher among those aged 20–24 (60.3%), 20–24 (57.1%), and 15–19 (53.6%) compared to those aged 40–44 (49.3%), 40–44 (45.8%), and 45–49 (39.5%) in 2017–2018, 2014, and 2011, respectively. This study found that the younger the women were, the greater the tendency to watch television at least once a week. Furthermore, those with complete secondary or higher education had the highest prevalence of watching television at least once a week at 75.5%, 80%, and 79.9%, respectively.

Furthermore, listening to the radio at least once a week was more prevalent among those aged 15–19 (3.9% and 6.8%) than among those aged 40–44 (0.9%) and 30–34 (4.4%) in 2017–2018 and 2011, but less prevalent among those aged 15–19 (4.3%) than those aged 40–44 (45.8%) in 2014 respectively. This study found that the younger the women were, the greater the tendency to listen to the radio at least once a week. Here, those with a complete secondary or higher education had the highest portion of watching television at least once a week at 5.4%, 5.6%, and 7.4%, respectively. In addition, accessing all three media at least once a week was higher among those aged 20–24 and 25–29 (0.5%), 25–29 (0.6%), and 15–19 (0.9%) compared to those aged 40–44 (0.1%), 35–39 (0.1%), and 40–44 and 45–49 (0.2%) in 2017–2018, 2014, and 2011, respectively. This study found that the younger the women were, the greater the tendency to listen to the radio at least once a week. Here, those with a complete secondary or higher education had the highest prevalence of watching television at least once a week at 1.9%, 1.7%, and 2.9%, respectively. In contrast, accessing none of the three media at least once a week was higher among those aged 45–49 (50.2%), 45–49 (52.8%), and 45–49 (58%) compared to those aged 20–24 (38.6%), 20–24 (41.4%), and 40–44 and 15–19 (43%) in 2017–2018, 2014 and 2011 respectively. This study found that the older the women were, the greater the tendency to access none of the three media at least once a week. Here, uneducated women had the highest prevalence of not accessing any of the three media at least once a week at 63%, 69.3%, and 70.5%, respectively.

Our findings are consistent with several research studies. For instance, mass media like TV and radio can impact people's perspectives on healthy lifestyles and family planning (70). Radio and television expose both sexes to current views and opinions on healthcare, family planning, and related matters. According to Fleischer (63), more people are using contraception because of what they see in the media. Furthermore, misperceptions about family planning and contraception are dispelled, and people are encouraged to use these methods by media depictions (71–73), indicating a substantial correlation between media exposure and modern birth control methods. The usage of modern methods of birth control increased with time among women who were less exposed to mass media. Even though this finding contradicts previous studies, we maintain our belief that media exposure significantly influences the use of contraceptive methods.

Studies have shown that the media is the main source of information regarding modern contraceptives’ benefits. Osman et al. (74) and Aslam et al. (75) found that media exposure increased modern contraception use. Ferrara et al. (76) found in Brazil that soap operas promote modest families, as individual choices diminish the birth rate. Television access reduced the birth rate in Indonesia, according to Dewi et al. (77). Barber and Axinn (78) discovered that mainstream media is linked to childbearing, small family preference, reduced son preference, and contraceptive tolerance in Nepal. In addition, women who were exposed to media about family planning were more likely to use contraception, according to research by Haq et al. (6). All three surveys found statistically significant levels of exposure to messages on family planning in various types of mass media. Islam et al. (39), Hussain (79), Kamal and Mohsena (80), Saleem and Pasha (81), and Mohsena and Kamal (54) all found comparable results in their studies on South Asia. Islam et al. (39) found that among Indian women, those who lacked exposure to mass media were less likely to use modern techniques. There was a concomitant shift in the ideal family size and the prevalence of contraception (82). The media's ability to understand and inspire couples, especially on delicate topics such as their reproductive means and objectives, may be a contributing factor to this finding. Findings from our study corroborate those of previous research (18, 83) that found that women with more formal education, occupations, and media exposure were more likely to use modern methods of contraception. Therefore, we can conclude that media exposure, especially television, is linked to current and future contraception use.

**Figure 7:**
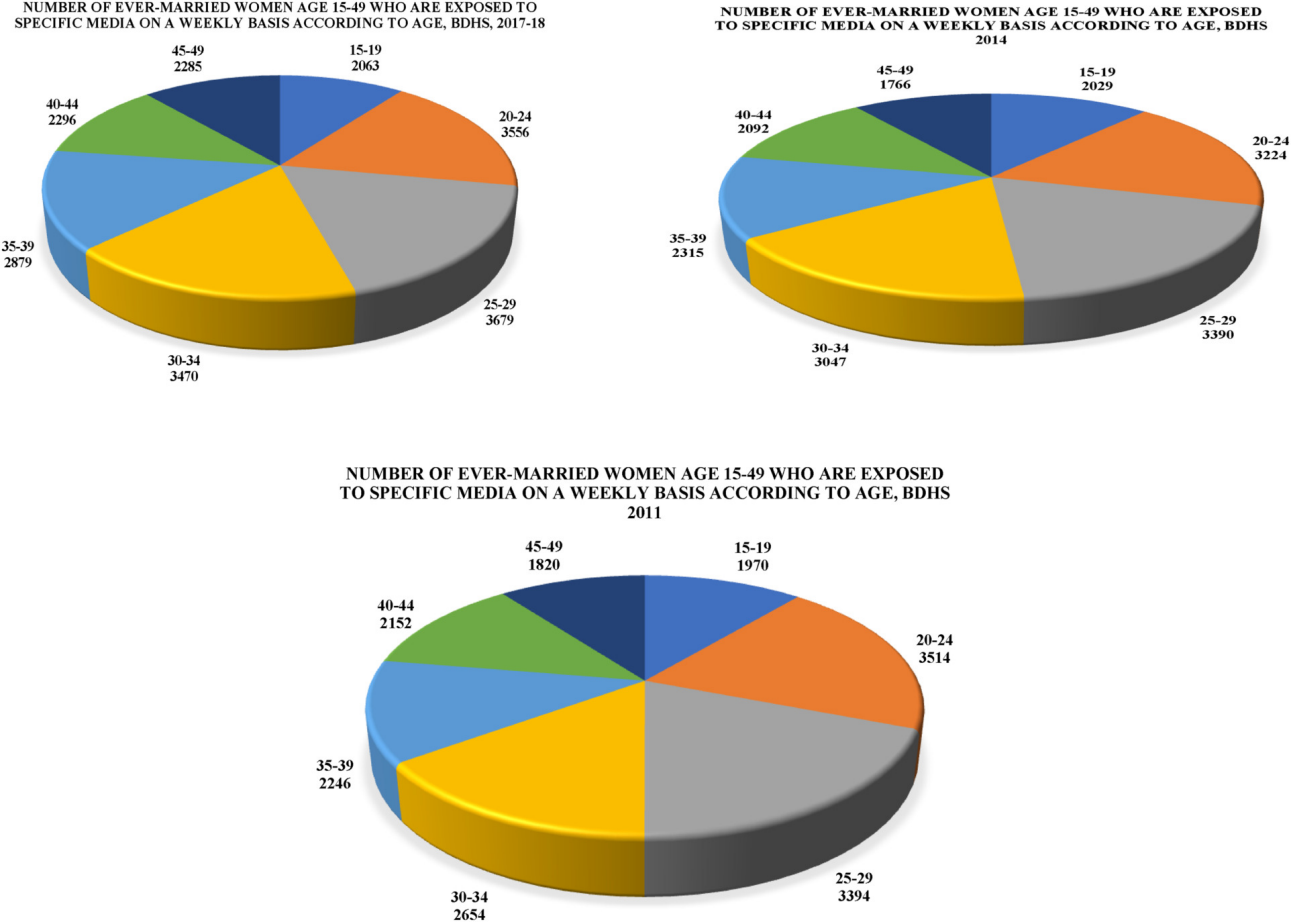
Exposure to mass media. Number of ever-married women aged 15–49 who were exposed to specific media on a weekly basis from the BDHS (24–26).

Figure 7 shows the number of ever-married women aged 15–49 who were exposed to specific media on a weekly basis from the BDHS (24–26). The 25–29 and 20–24 age groups had the highest number of women with 3,679, 3,390, and 3,514 in 2017–2018, 2014, and 2011, respectively. The lowest numbers belonged to the 45–49 age group with 2,285, 1,766, and 1,820, respectively. Thus, the figure depicts that the older the women, the lower the number of those exposed to media about their contraception practices on a weekly basis.

Despite Bangladesh's achievement in reducing the birth rate, reproductive-age women must use contraception. Bangladesh has a long way to go to reach the desired CPR of above 70% (84). Bangladesh has extensively studied the factors that affect contraceptive use (46, 84, 85). The majority of this study used nationally representative sample surveys. Comparative studies using national surveys from different years can reveal the factors that affect contraceptive use in Bangladesh. During the past two decades, there has been a significant surge in the usage of contraception worldwide, especially in poorer nations. The increasing adoption of contraceptive technologies in underdeveloped nations has resulted in a 40% decrease in unwanted pregnancies and maternal deaths. Furthermore, research carried out in Bangladesh (86) presented data indicating that the incidence of unintended pregnancies was greater (33%) among women who used contraceptives before their most recent pregnancy compared to women (23%) who did not use any reproductive methods. In general, the findings of these studies suggest that women's autonomy essentially facilitates their greater role in decision-making related to contraceptive use, and makes women more capable to overcome barriers related to contraceptive use such as resisting family and social pressure of having more children, avoiding misconceptions about contraception, and taking initiatives to deal with any side effects of using contraception (87).

Overall, the results of this research indicated a positive correlation between maternal age, level of education, and media exposure with contraceptive usage in Bangladesh. One study revealed that women aged 35 and older have a reduced frequency of contraceptive use due to their heightened apprehension about perceived health issues (88). Adolescent women use contraception more than older women because they are more aware of preventing unwanted pregnancies and want to postpone childbearing (89). Women with higher education are more likely to utilize contraception than women with lower levels of education because they are more aware of the detrimental effects of having more children on the health of both mothers and children. It is expected of spouses to limit the number of children their wife has through family planning. According to Kamal et al. (90), there is evidence that women broach the topic of contraception with their spouses, but that men ultimately make the choice because they are the ones who provide the money. Due to the power disparities in many relationships, women may experience fear of violence when family planning or the use of contraceptives is discussed. As a result, this study's findings expand on our understanding of the role that decision-making authority plays in both Bangladesh's population and the current prevalence of contraception.

### Strengths and drawbacks of the study

The utilization of nationally representative data allows for a detailed examination of the findings, which is the principal strength of this study. This study provided a thorough explanation of the modern and traditional methods of birth control used by Bangladeshi women. However, this study has certain drawbacks. Men's perceptions of their female partners’ acceptance of barrier methods of contraception or any other contemporary technique of contraception were excluded. This study started by comparing the results to those of other rich and poor countries. However, because this study used secondary datasets, we could not include many important factors due to data limitations. The study did not ask about the interviewees’ family situations. In addition, the study failed to consider whether couples live in a nuclear or shared family structure, which could have limited the generalizability of the results. Not including relevant variables in the datasets also meant that the study's findings did not account for how healthcare access, family planning programs, and public views on contraception have evolved over time. Notwithstanding these drawbacks, the study offers insightful information about the patterns and factors influencing Bangladeshi women's adoption of both traditional and modern forms of contraception, laying the groundwork for further investigations and policy creation.

## Conclusion and recommendations

Our review provides a substantial body of evidence on contraceptive behavioral practices and media influence among women in Bangladesh based on the effects of age and education. Since its independence, Bangladesh has seen varied contraceptive behavioral practices associated with factors like age and education, which have played a significant role. Age and education influence the autonomy of contraception and the choice of using a specific method, which plays a vital role in the reproductive health of women. When considering women's autonomy in decision-making about contraception practices aimed at slowing rapid population growth, prioritizing women's empowerment becomes crucial. Mass media should be active and try to find out the problems faced by women with the cooperation of the health workers, so that they can express their views and get age-appropriate contraception suggestions from the health workers. The number of educational facilities should be increased so that women can gather relevant information about contraception and hence, unwanted pregnancies will be reduced. Finally, as it is difficult to disaggregate the effectiveness of individual techniques, we recommend further in-depth research to understand which techniques are especially effective for women according to their age category. In particular, we recommend performing an additional in-depth study on solutions to problems, techniques for improving performance, and interventions providing social support. It is likely that the only method of birth control that will be effective is a mix of several behavioral routines. To effectively promote women's empowerment in terms of health, it is necessary to employ subtle communication strategies that encourage women to participate in the process of planning and determining the size of their family, while simultaneously dispelling any negative beliefs or misunderstandings that may exist regarding modern methods of contraception. As a result, this study has some implications for public policy, including the provision of information that is necessary to comprehend the adverse effects of overpopulation on a country like Bangladesh, the implementation of a face-to-face communication program, the provision of family planning education that is based in institutions, and the guarantee of exposure to electronic media. To boost the nation's usage of contraceptives, health promotion and educational initiatives should focus on workplaces, schools, mosques, churches, and temples.

## Data Availability

The raw data supporting the conclusions of this article will be made available by the authors, without undue reservation.
